# Multi-source COVID-19 surveillance in Fujian, China: epidemiological features of two epidemic waves and public health implications

**DOI:** 10.3389/fpubh.2026.1857657

**Published:** 2026-10-29

**Authors:** Jiawei Lin, Senshuang Zheng, Zhifang Zhang, Jingjing Cai, Cailin Chen, Yuedong Lin, Jianming Ou, Hansong Zhu, Wenjing Ye, Shenggen Wu

**Affiliations:** 1 Fujian Center for Disease Control and Prevention, Fujian Provincial Key Laboratory of Zoonosis Research, Fuzhou, China; 2 School of Public Health, Fujian Medical University, Fuzhou, China; 3 Xiamen Center for Disease Control and Prevention, Xiamen City, Fujian Province, China; 4 Chinese Field Epidemiology Training Program, Chinese Center for Disease Control and Prevention & Chinese Academy of Preventive Medicine, Beijing, China

**Keywords:** China, COVID-19, epidemiological characteristics, multi-source surveillance, multi-wave epidemic, public health implications

## Abstract

**Objective:**

Using Fujian’s multi-source Coronavirus Disease 2019 (COVID-19) surveillance system, this study compared epidemiological features and indicator associations of two successive epidemic waves to optimize epidemic surveillance and prevention strategies.

**Methods:**

Multi-source surveillance data were collected from week 49 of 2022 (the week of December 7, 2022, when the 10 optimization measures were issued) to week 29 of 2023 (July 17–23, 2023) in Fujian Province, including reported COVID-19 cases, fever clinic visits, population nucleic acid testing, sentinel hospital surveillance, hospitalized severe cases, and viral variants. Descriptive epidemiology, Spearman correlation, and lagged cross-correlation analyses were adopted for data evaluation.

**Results:**

The first wave showed a sharp peak pattern, with most indicators peaking around weeks 51–52/2022 and reported cases peaking at week 1/2023; the second wave exhibited a gradual peak at week 21/2023. Peak levels of the second wave were 10.7–99.9% lower across indicators, although the markedly reduced nucleic acid testing volume and changed surveillance conditions warrant cautious interpretation of cross-wave comparisons. The interval between the two epidemic peaks was about 5 months, and severe cases consistently lagged infection peaks by approximately 2 weeks. Indicators were strongly correlated, with the highest correlation between sentinel and nucleic acid positivity rates (rs = 0.92, *p* < 0.001); lagged cross-correlation analysis indicated that these two indicators moved essentially in synchrony. The XBB variant took approximately 4 weeks from initial local detection to predominance alongside the second epidemic peak.

**Conclusion:**

The two observed epidemic peaks were approximately 5 months apart, and multi-source surveillance indicators showed high temporal concordance. However, lagged cross-correlation indicated that the indicators moved essentially in synchrony, so sentinel surveillance should be regarded as a complementary monitoring tool rather than a proven early-warning measure. The XBB variant reached predominance approximately 4 weeks after initial local detection in this episode, a descriptive observation that cannot be generalized as a validated predictive tool. These findings facilitate multi-wave epidemic preparedness and surveillance improvement.

## 1 Introduction

The coronavirus disease 2019 (COVID-19) pandemic has featured recurrent global epidemic waves. Surveillance data suggest that epidemic peaks occur approximately every 4–5 months, driven mainly by immune escape of viral variants, changes in pathogenicity, and natural waning of population immunity (1–3).

Following the optimization of COVID-19 prevention and control measures in China on December 7, 2022 (4), the first wave of epidemic spread occurred successively across regions. Fujian Province reached its first wave peak dominated by the BA.5.2 variant at week 52 of 2022 (5). Since 2023, the XBB variant has gradually spread locally in Fujian and become the dominant strain due to its strong immune escape ability (6, 7), leading to the second wave peak. Nevertheless, the dynamic epidemiological characteristics and synchronous variation of multi-source surveillance indicators across these two consecutive local waves have not been fully clarified.

Most existing domestic studies merely describe epidemiological characteristics of single COVID-19 waves or conduct separate analyses of individual surveillance indicators. Continuous comparative studies based on standardized, unified multi-source surveillance data covering two consecutive local Omicron epidemic waves are still lacking. Previous studies have analyzed single-wave epidemiological characteristics using Fujian’s multi-source surveillance system (5), but few studies have systematically compared wave-specific epidemic progression, stage-specific correlation heterogeneity of surveillance indicators, and inherent limitations of non-specific monitoring indexes across different epidemic phases (1, 3). Biases in routine influenza surveillance indicators used to monitor infection incidence and recommendations for improvement (8).

Elucidating the nonlinear evolutionary characteristics of dual-wave epidemics and the dynamic synchronous relationship of multi-source surveillance indicators is urgently required to support real-time epidemic surveillance, rational advance deployment of medical resources and anticipation of variant-induced epidemic rebounds. This study used continuous surveillance data from week 49 of 2022 to week 29 of 2023 to compare two waves, verify indicator correlations, analyze transmission dynamics, and provide evidence for public health preparedness and surveillance optimization. Specifically, it further explored the phase-specific nonlinear variation of multi-index associations and illustrated the practical limitations of non-specific respiratory surveillance indicators, aiming to provide targeted optimization strategies for regional post-pandemic surveillance systems.

## 2 Materials and methods

### 2.1 Study setting and data sources

This study was conducted in Fujian Province, eastern China. De-identified and aggregated weekly surveillance data were collected from week 49 of 2022 (December 7–13, 2022) to week 29 of 2023 (July 17–23, 2023), covering the full ascending, peak, and descending phases of two COVID-19 waves. Data were obtained from the Fujian Provincial COVID-19 Multi-Source Surveillance System, with detailed items listed below:

1. Nationally notifiable COVID-19 cases reported by all medical institutions across Fujian through the national infectious disease surveillance reporting system.
2. Fever clinic surveillance data from 355 secondary and above hospitals plus 1,107 community health centers/township health clinics, including total outpatient visits and COVID-19 positive test counts. All data were uniformly aggregated and reported at the provincial level, with no independent records of individual specimen submission volumes.
3. Population nucleic acid testing data uniformly administrated by Fujian Provincial CDC, covering community free screening and voluntary testing at designated hospitals across all prefecture-level cities. Raw weekly data were uploaded to the provincial infectious disease information platform, aggregated with unified statistical criteria, and the positivity rate was calculated as weekly positive count/total weekly tested population ×100%. The tested population comprised community screening and voluntary/hospital-based testing rather than a representative sample of the general population; accordingly, this indicator reflects the test-positive proportion among tested individuals and cannot be directly equated to true population infection prevalence. Notably, the scale of population nucleic acid testing decreased dramatically between the two epidemic waves, from a peak of approximately 7.49 million tests in week 51/2022 to approximately 78,000 tests at the peak of the second wave (week 21/2023); shifts in testing policy and availability, healthcare-seeking behaviour, reporting practices, and pre-existing population immunity across the two waves may thus contribute to observed inter-wave differences, and cross-wave comparisons of indicator levels should be interpreted cautiously. Only province-level aggregated weekly data without any individual personal identifiers were included in the analysis.
4. Sentinel hospital surveillance from 15 national and 3 provincial influenza sentinel sites, including the proportion of influenza-like illness (ILI) and the positive rates of COVID-19 and influenza among ILI specimens;
5. Weekly counts of hospitalized COVID-19 patients and severe cases collected from all 355 secondary and above hospitals.
6. Severe Acute Respiratory Syndrome Coronavirus 2(SARS-CoV-2) whole-genome sequencing results collected in accordance with Fujian’s provincial variant surveillance protocol, with specimen sources including Fuzhou No.2 Hospital, the First Affiliated Hospital of Xiamen University, Quanzhou First Hospital and other designated sentinel institutions.

### 2.2 Definitions

Fever clinic positive diagnosis ratio (%) = positive COVID-19 tests/total fever clinic visits × 100%. ILI: body temperature ≥38 °C with cough or sore throat.

$\begin{array}{c} \mathrm{ILI}\;\text{proportion}\;(\%)=\mathrm{ILI}\;\text{cases}/\text{total outpatient}\;\\ \;\;\;\text{and emergency visits}\times 100\% \end{array}$

Epidemic stage definitions: Onset week: first week of continuous increase after a periodic low. Peak week: the week with the highest value of each indicator in one epidemic wave. Peak cycle: weeks from onset week to peak week. Sharp peak: rapid rise and fall with a narrow peak shape. Gradual peak: slow rise and fall with a broad peak shape.

### 2.3 Statistical analysis

Data were organized using Excel 2019 and analyzed using R software (version 4.4.1). Descriptive epidemiological methods were applied to systematically compare the epidemic characteristics and indicator variation patterns between the two successive COVID-19 waves. Spearman correlation analysis was performed to evaluate indicator relationships across the full study period, stratified by epidemic wave, and within four epidemic phases (ascending and descending phases of each wave). Because direct correlations between time series can be inflated by shared secular trends and temporal autocorrelation, lagged cross-correlation analysis was additionally performed between the population nucleic acid positivity rate and the sentinel hospital COVID-19 positivity rate: the two weekly series were shifted by lags ranging from −4 to +4 weeks, and the Spearman correlation coefficient was calculated for each lag to examine potential lead–lag relationships. Phase-stratified correlation results based on small numbers of weekly observations were interpreted descriptively and with caution. A *p*-value <0.05 was considered statistically significant. To facilitate replication, the aggregated weekly dataset, analytical parameters, and R analysis scripts are available from the corresponding authors upon reasonable request.

## 3 Results

### 3.1 Notifiable legally reported cases

The peak magnitude and epidemic peak shape of reported cases differed remarkably between the two COVID-19 waves.

For the first wave (week 49 of 2022 to week 16 of 2023), reported cases started increasing at week 49/2022 and reached a peak of 16,513 at week 1/2023 after four consecutive rising weeks. Case values fell to 92 by week 16/2023 over around 20 weeks, with a minor rebound after school reopening and a sharp peak feature (Figure 1).

**Figure 1:**
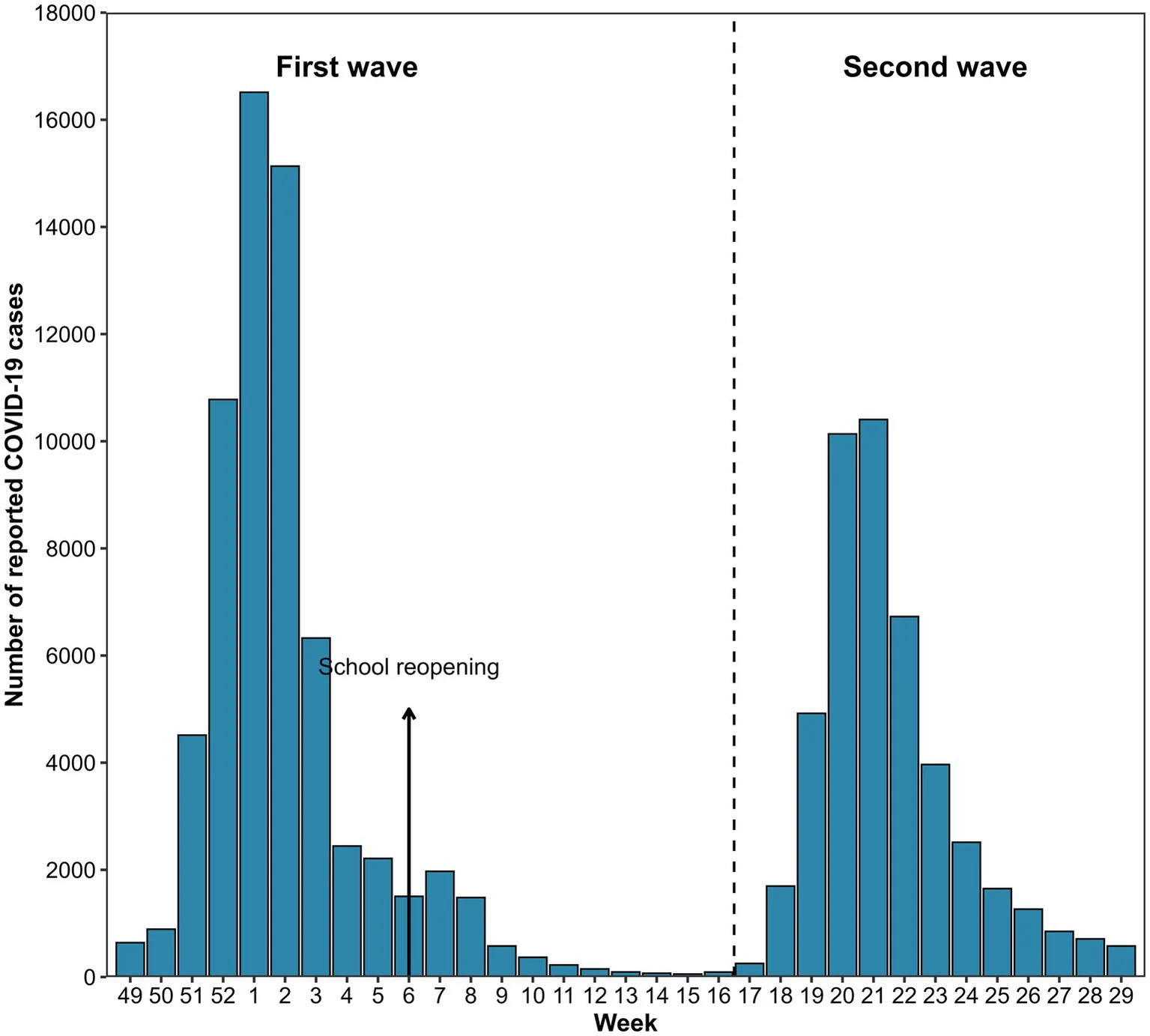
Dynamic changes in the number of directly reported COVID-19 cases in Fujian Province, China, from week 49 of 2022 to week 29 of 2023.

For the second wave (week 17–29/2023), case numbers rose from week 17/2023 and peaked at 10,405 in week 21/2023, then gradually reduced to 579 by week 29/2023 across roughly 12 weeks with a gradual peak shape without returning to the first-wave baseline (Figure 1). The peak case count of the second wave was 37.0% lower than that of the first wave.

Distinct shifts in age and gender composition were observed across the two epidemic cohorts. In Wave 1, males accounted for 57.03% and females 42.97% of total cases. Patients aged ≥60 accounted for 37.82% of male cases and 24.32% of female cases, while children aged 0–14 made up below 6% of all cases for both genders. In Wave 2, male and female cases were nearly balanced at 49.70 and 50.31%, respectively. The proportion of patients aged ≥60 decreased obviously for both sexes; the proportion of children aged 0–14 rose, and adults aged 15–59 became the predominant population (Figure 2). The epidemic population shifted from an older-adult-predominant structure in the first wave toward a younger composition in the second wave.

**Figure 2:**
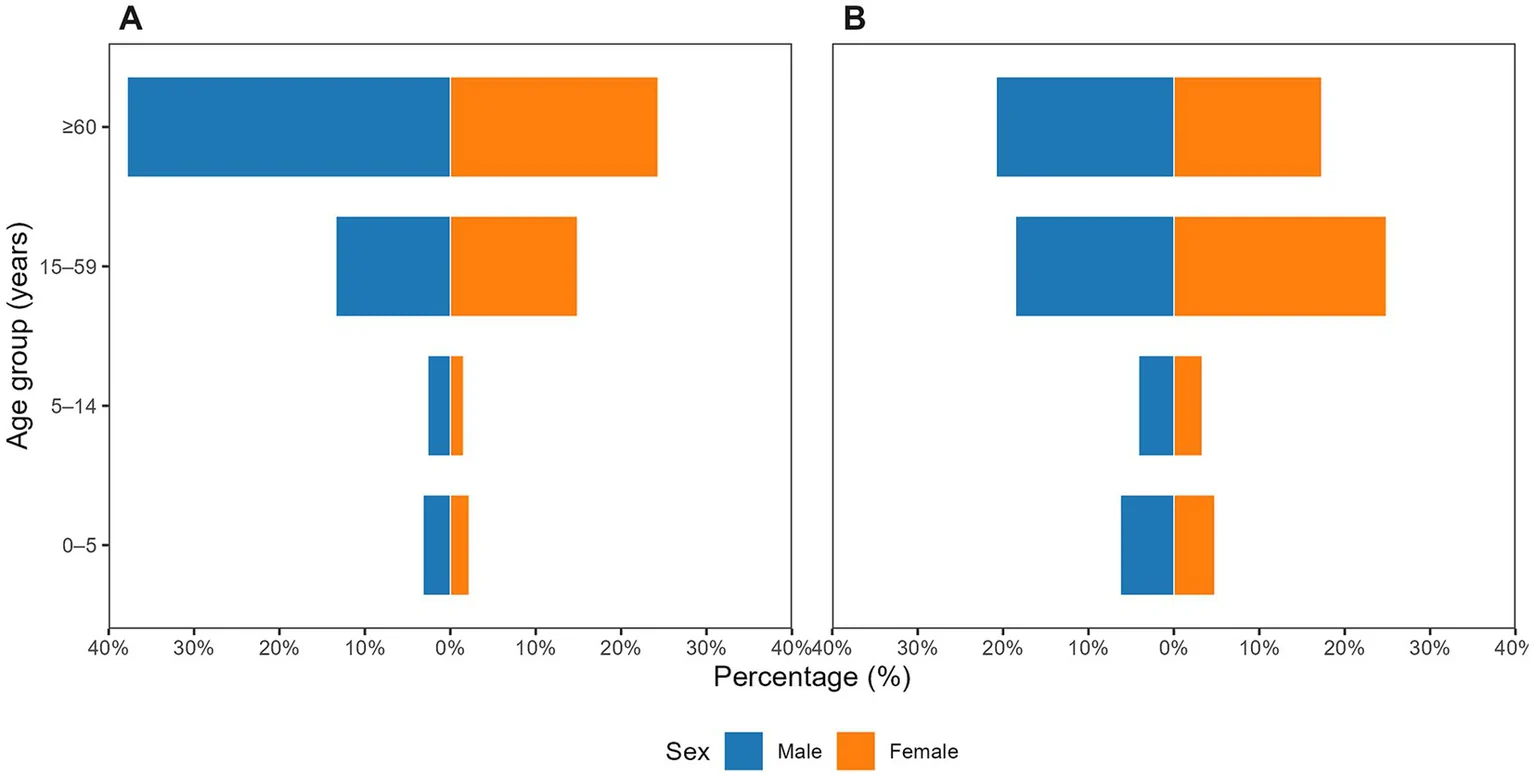
Age-sex distribution pyramid of cases in two epidemic waves. **(A)** First epidemic wave spanning week 49 of 2022 to week 16 of 2023; **(B)** Second epidemic wave spanning week 17 to week 29 of 2023.

### 3.2 Fever clinic surveillance

Obvious discrepancies in peak magnitude and evolving patterns of fever clinic indicators were observed between two epidemic waves. For the first wave, relevant indicators began to rise at week 49/2022. The peak positive diagnosis ratio hit 34.2% at week 51/2022, and total fever clinic visits peaked at 6.069 million person-times at week 52/2022; a minor rebound occurred after school resumption, corresponding to a sharp peak shape (Figure 3). During the second wave, indicators increased starting from week 17/2023. The positive diagnosis ratio peaked at 5.4% (week 20/2023), while outpatient visits reached the maximum value of 1.074 million person-times at week 21/2023, featuring a gradual peak without returning to pre-epidemic baseline (Figure 3). Compared with the first wave, the peak values of fever clinic visits and positive diagnosis ratio of the second wave decreased by 82.3 and 84.2%, respectively.

**Figure 3:**
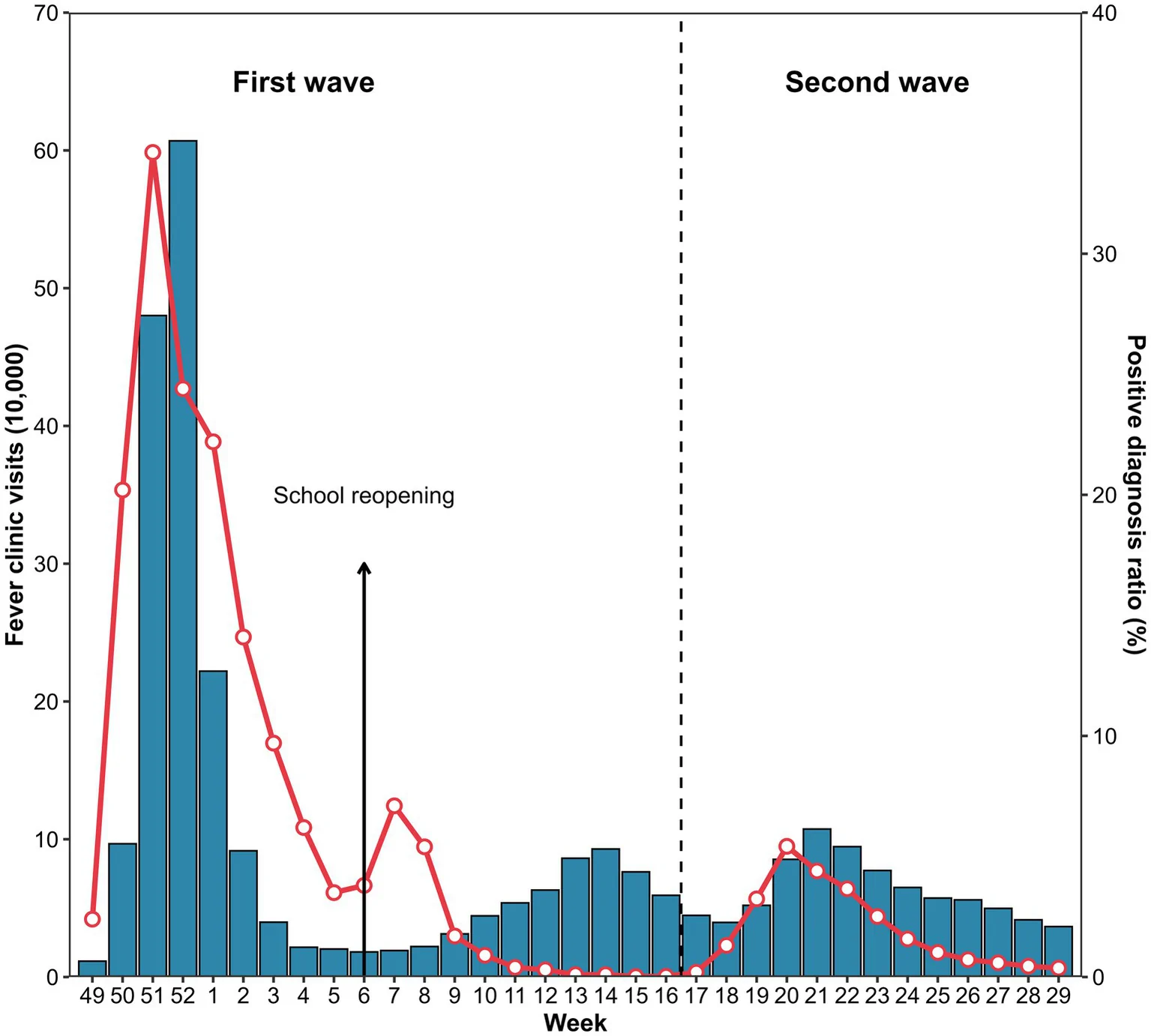
Dynamic changes in fever clinic visits and COVID-19 positive diagnosis ratio (%) in Fujian Province, China, from week 49 of 2022 to week 29 of 2023.

### 3.3 Population nucleic acid surveillance

Distinct differences in testing scale and positivity peaks existed across the two epidemic waves. For the first wave, nucleic acid-related indicators started rising at week 49/2022. Total testing volume peaked at 7.489 million at week 51/2022, and the population positivity rate reached 49.2% at week 52/2022. A small rebound in positivity was seen after school reopening, forming a sharp peak with high testing volume (Figure 4). For the second wave, indicators increased from week 17/2023. Testing quantity and positivity rate peaked synchronously at week 21/2023 (78,000 tests and 35.6%), showing a gradual peak alongside remarkably reduced testing scale (Figure 4). The second-wave peak testing volume and positivity declined by 89.6 and 27.6% relative to the first-wave peak values.

**Figure 4:**
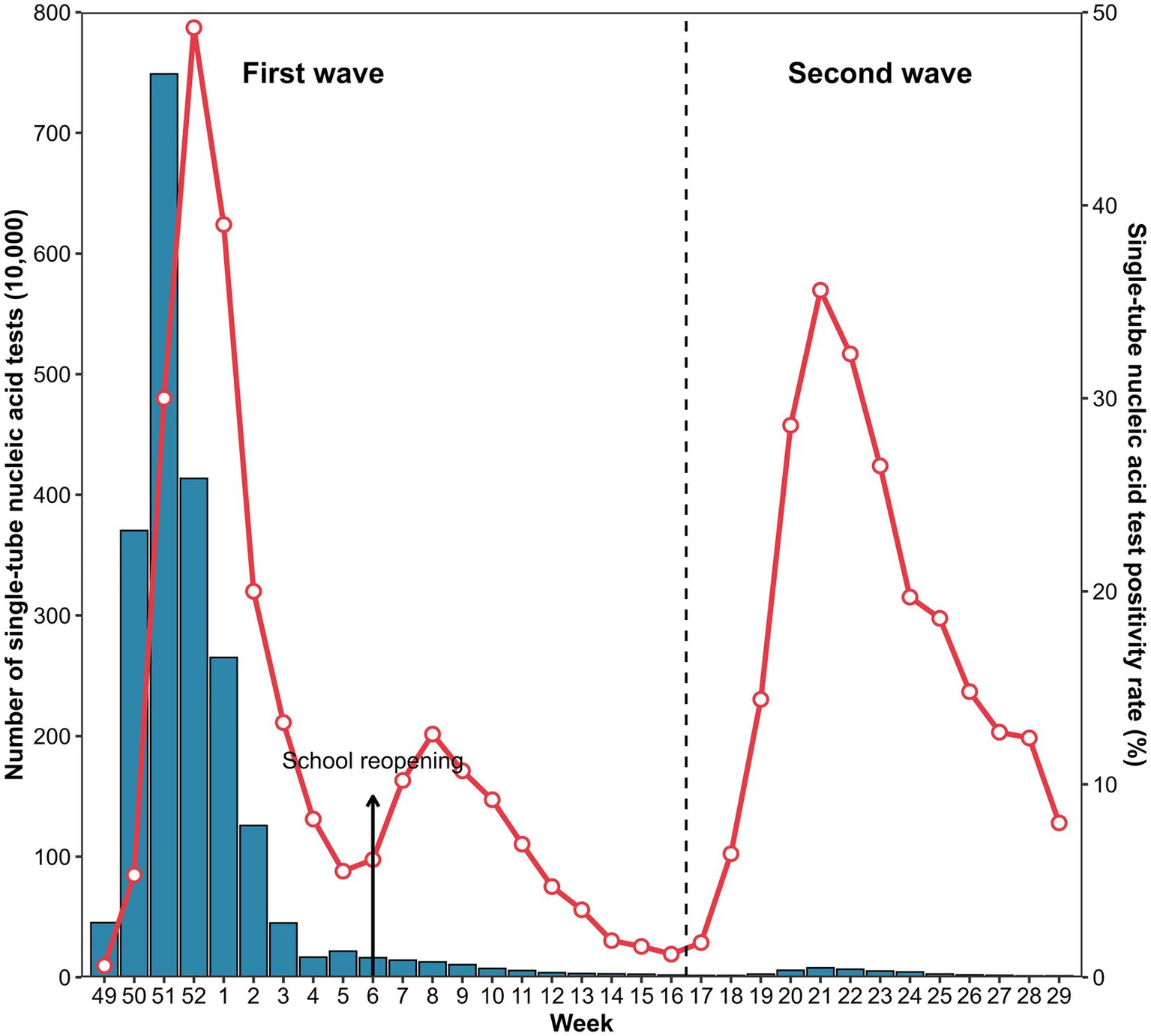
Dynamic changes in the number of single-tube nucleic acid tests and population nucleic acid positivity rate (%) in Fujian Province, China, from week 49 of 2022 to week 29 of 2023.

### 3.4 Sentinel hospital surveillance

The changing trends of ILI proportion and pathogen positive rates differed obviously between the two epidemic waves. For the first wave, sentinel surveillance indicators began rising at week 49/2022. COVID-19 positivity peaked at 63.8% (week 52/2022), while the maximum ILI proportion was 19.12% at week 51/2022; influenza positivity stayed at zero during the outbreak period, and influenza activity increased in the inter-wave low-incidence stage (Figure 5). For the second wave, indicators increased starting at week 17/2023. COVID-19 positivity peaked at 57.0% (week 21/2023) and ILI proportion peaked at 12.77% (week 20/2023), with low influenza prevalence across the whole phase (Figure 5). Peak values of COVID-19 positivity and ILI proportion were both lower in the second epidemic wave.

**Figure 5:**
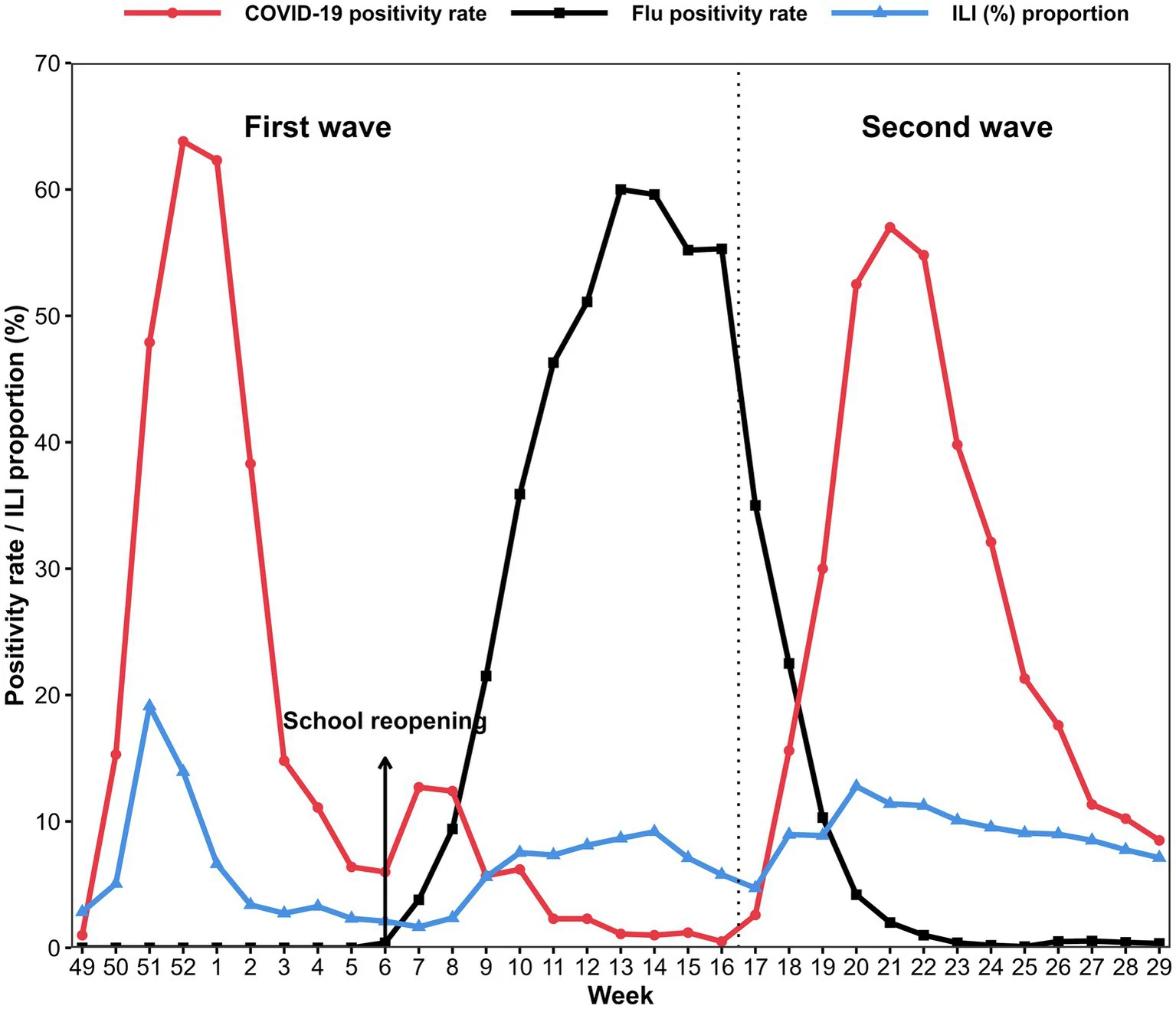
Dynamic changes in influenza-like illness (ILI) proportion (%), COVID-19 positivity rate (%), and influenza positivity rate (%) at sentinel hospitals in Fujian Province, China, from week 49 of 2022 to week 29 of 2023.

### 3.5 Correlation of multi-source indicators

Overall and wave-stratified correlation features of four surveillance indicators presented distinct discrepancies. Spearman-based full-cycle correlation analysis revealed the strongest correlation between population nucleic acid positivity and sentinel COVID positivity (rs = 0.92, *p* < 0.001, Figures 6A,B). Moderate correlation existed between population nucleic acid positivity and fever clinic positive ratio (rs = 0.62, *p* < 0.001), whereas a weak positive correlation was found between population nucleic acid positivity and ILI proportion (rs = 0.52, *p* < 0.05); sentinel COVID positivity was strongly correlated with fever clinic positive ratio (rs = 0.72, *p* < 0.001). To examine potential lead–lag relationships, lagged cross-correlation analysis was performed between the population nucleic acid positivity rate and the sentinel hospital COVID-19 positivity rate (Figure 6E). The maximum correlation was observed at a lag of −1 week (i.e., sentinel positivity leading population positivity by one week; rs = 0.931, *p* < 0.001), which was only marginally higher than the contemporaneous correlation (rs = 0.925 at lag 0), and correlations declined rapidly at lags beyond ±1 week (rs = 0.658 at lag −2; rs = 0.674 at lag +1). Given the strong temporal autocorrelation in both series and the negligible difference between the peak and contemporaneous correlations, these results indicate that the two indicators moved essentially in synchrony, without a consistent lead–lag (early-warning) advantage in this dataset.

**Figure 6:**
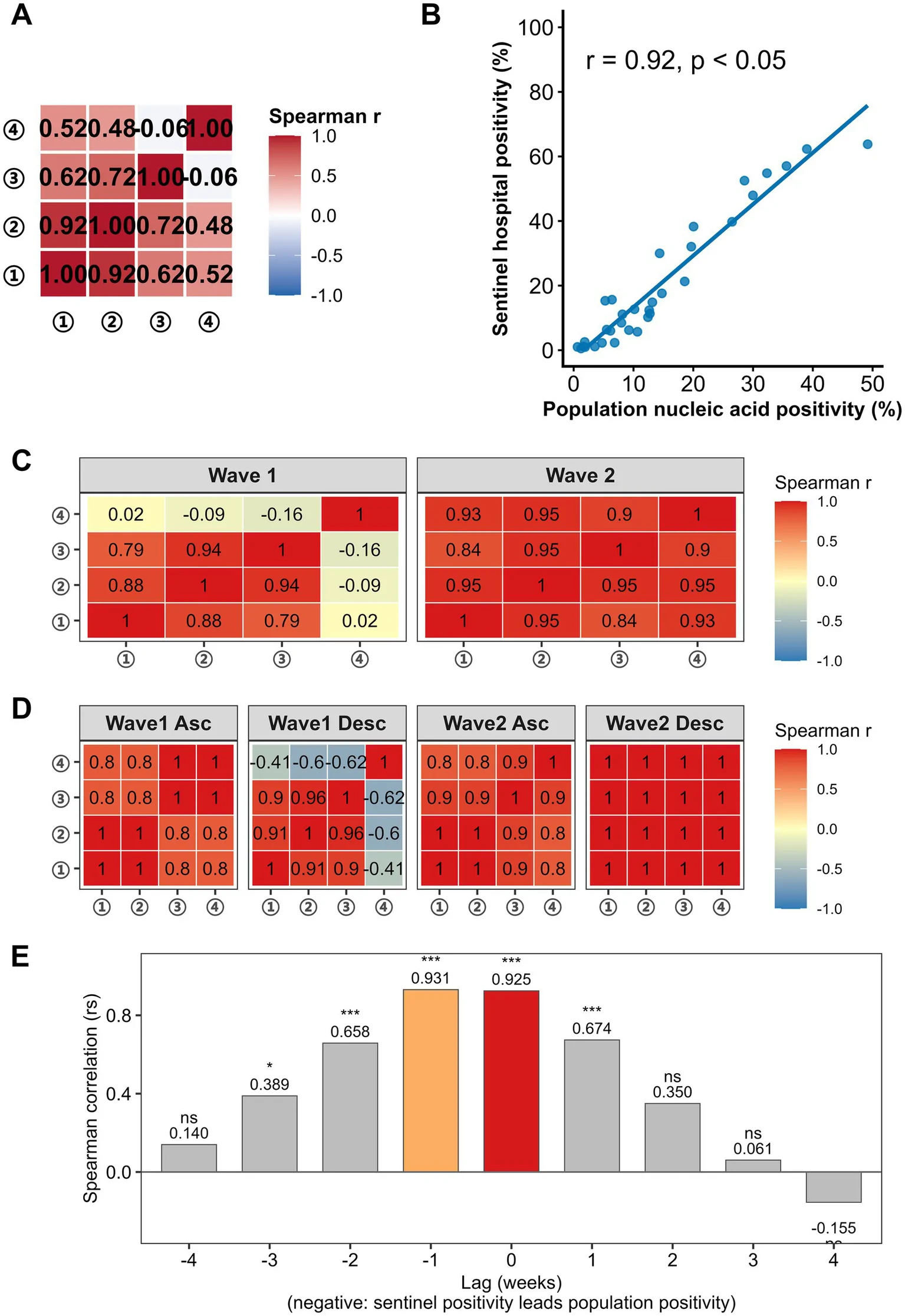
Correlation analysis of multi-source COVID-19 surveillance indicators in Fujian Province, China. **(A)** Spearman correlation heatmap of four indicators: ① population nucleic acid positivity rate; ② sentinel hospital COVID-19 positivity rate; ③ fever clinic positive diagnosis ratio; ④ ILI proportion. **(B)** Scatter plot and linear regression between population nucleic acid positivity rate and sentinel hospital COVID-19 positivity rate. **(C)** Wave-stratified correlation results. **(D)** Four-phase stratified correlation results. **(E)** Lagged cross-correlation between population nucleic acid positivity rate and sentinel hospital COVID-19 positivity rate.

Stratified by epidemic wave, three COVID-related indicators were strongly correlated (rs = 0.79–0.88) in Wave 1, while ILI proportion had near-zero or weak negative associations with these three indices (rs = −0.16 ~ 0.02); all four indicators showed strong positive correlations ranging from 0.84 to 0.95 in Wave 2 (Figure 6C). Four epidemic phases exhibited obvious differentiated correlation results across indicators.

All indicators were strongly positively correlated (rs = 0.80–1.00) during the ascending phases of both waves. In Wave 1 Descending stage, ILI proportion was moderately negatively correlated with three COVID-related indicators (rs = −0.41 ~ −0.62), though the three COVID indices maintained high inter-correlation (rs = 0.90–0.96). Every pairwise correlation coefficient approximated 1.00 in the descending phase of Wave 2 (Figure 6D). Of note, each epidemic phase comprised only a limited number of weekly observations; correlation coefficients from these phase-stratified analyses should therefore be interpreted with caution, as near-perfect correlations may partly reflect the small sample sizes and shared temporal trends rather than stable underlying associations.

### 3.6 Temporal patterns

The temporal characteristics of infection-related indicators were highly consistent between the two waves, both following the pattern: fever clinic indicators peaked first, followed by simultaneous peaks in population nucleic acid and sentinel hospital indicators. The fever clinic positive diagnosis ratio peaked 1 week earlier than the other two indicators, while population nucleic acid positivity rate and sentinel hospital COVID-19 positivity rate peaked simultaneously in both waves (Figure 7).

**Figure 7:**
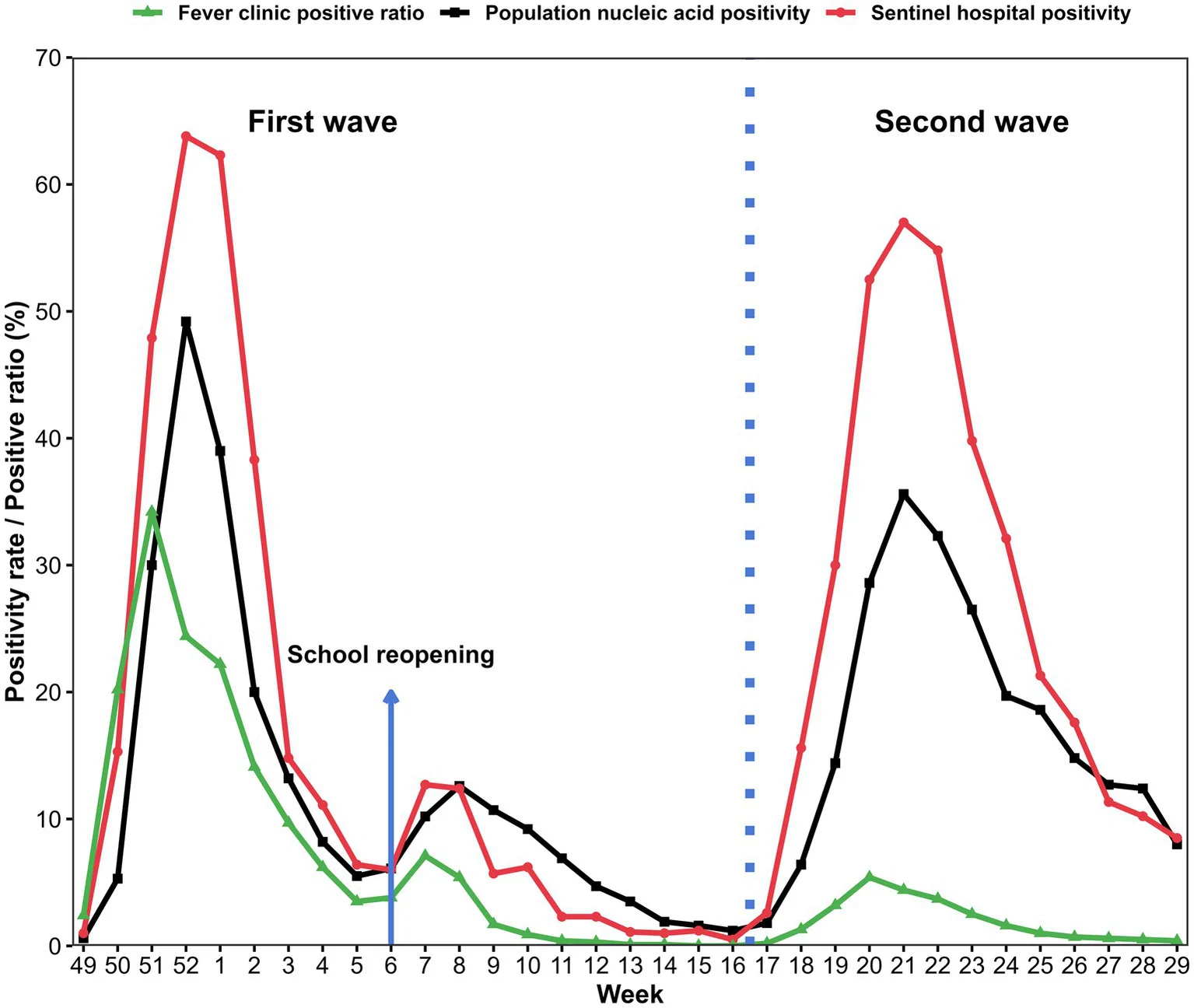
Dynamic changes in population nucleic acid positivity rate (%), sentinel hospital COVID-19 positivity rate (%), and fever clinic positive diagnosis ratio (%) in Fujian Province, China, from week 49 of 2022 to week 29 of 2023.

This pattern aligns with the high temporal synergy among indicators, indicating that multi-source indicators can mutually validate and complement each other for routine surveillance. However, the near-simultaneous peaking of these indicators also implies that none exhibited a consistent leading advantage over the others in this dataset; their early-warning value should therefore not be over-interpreted. The time from onset to peak for infection indicators was 2–3 weeks in the first wave and extended to 3–4 weeks in the second wave, indicating a slower epidemic progression consistent with the evolution from a sharp peak pattern to a gradual peak pattern.

### 3.7 Hospitalization and severe cases

The peak values and peak occurrence time of hospitalized and severe cases differed greatly between two epidemic waves. During the first wave, hospitalized cases peaked at 274,460 person-times and severe cases peaked at 28,487 person-times in week 2 of 2023, approximately 2–3 weeks after the peak of infectious surveillance indicators. Both indicators dropped rapidly after peaking; severe cases fell to zero by week 7/2023 and hospitalized cases reduced to 399 person-times by week 16/2023 (Figure 8). In the second wave, hospitalized cases peaked at 14,250 person-times (week 22/2023) and severe cases peaked at 17 person-times (week 23/2023), 1–3 weeks following the peak of infection indicators (Figure 8). Compared with the first wave, peak hospitalized cases and severe cases of the second wave dropped by 94.9 and 99.9% separately.

**Figure 8:**
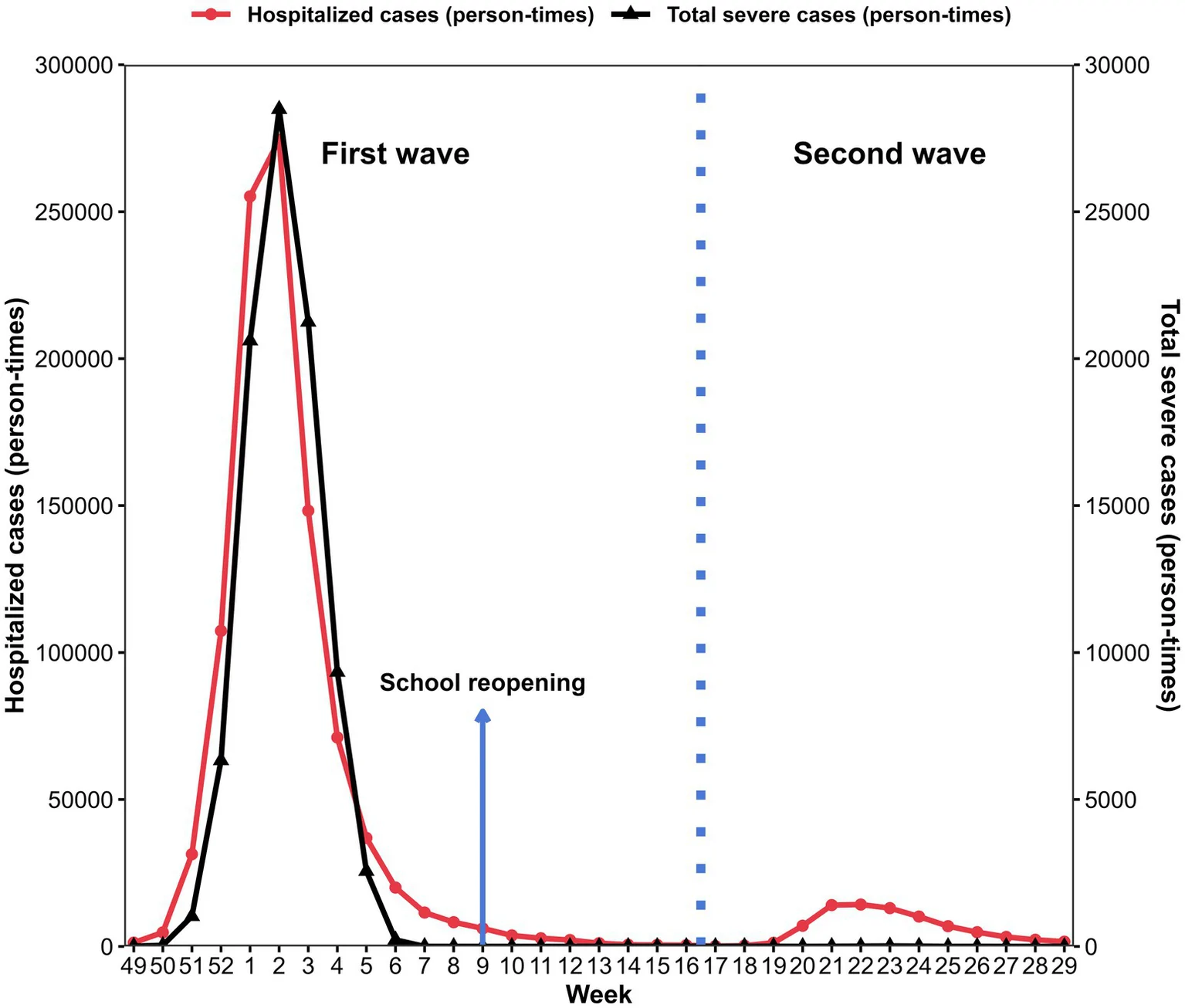
Dynamic changes in the number of hospitalized COVID-19 cases (person-times) and total severe/critical cases (person-times) in Fujian Province, China, from week 49 of 2022 to week 29 of 2023.

### 3.8 Viral variant dynamics

The XBB variant showed an approximately four-week interval from initial local detection to becoming the dominant epidemic strain in this local episode. BA.5.2 and BF.7 prevailed during the first epidemic wave. XBB was initially found in imported specimens at week 6/2023 and made up over half of imported strains at week 9/2023. The first local XBB was detected at week 13/2023, which exceeded 50% of local isolates at week 17/2023 and turned into the dominant strain when the second epidemic peaked at week 21/2023.

### 3.9 Comparison of two waves

Core surveillance indicators shifted from sharp-peak to gradual-peak characteristics with universally reduced peak magnitudes across the two waves (Table 1); given the substantial reduction in nucleic acid testing volume and the changes in testing policy and reporting practices between the two waves, these comparisons should be interpreted as reflecting a combination of epidemiological and surveillance-related changes. The peak-to-peak interval of core infection indicators spanned 21 weeks (around 5 months). Indicators required 2–3 weeks to reach peaks over a 20-week epidemic period in the first wave, whereas peak-delaying extended to 3–4 weeks with roughly 13 weeks of epidemic duration in the second wave; all indicator peak values declined by 27.6–99.9% (Table 1). Minor peak shifts of 1–2 weeks existed among distinct indicators, while overall epidemic trends kept consistent for two waves (Table 1).

**Table 1:** Comparison of key surveillance indicators for the first and second COVID-19 waves in Fujian Province, China.

Surveillance dimension	First wave^a^ (Week 49 of 2022–Week 16 of 2023)			Second wave^b^ (Week 17 of 2023–Week 29 of 2023)		
	Onset week	Peak week	Peak level	Onset week	Peak week	Peak level
Directly reported cases (*n*)	49	1	16,513	17	21	10,405
Fever clinic positive diagnosis ratio (%)	49	51	34.2	17	20	5.4
Population nucleic acid positivity rate (%)	49	52	49.2	17	21	35.6
Sentinel hospital COVID-19 positivity rate (%)	49	52	63.8	17	21	57.0
Hospitalized cases (person-times)	49	2	274,460	18	22	14,250
Severe cases (person-times)	49	2	28,487	19	23	17

*Sharp peak pattern*: rapid rise to infection peak within 3 weeks and severe peak within 5 weeks after onset; high peak level and strong transmission intensity, with a small rebound during the school reopening period. ^b^*Gradual peak pattern*: slow rise to infection peak within 4 weeks and severe peak within 6 weeks after onset; significantly lower peak than the first wave, slow decline after peak and not returning to the baseline of the first wave.

## 4 Discussion

This study systematically analyzed the epidemiological characteristics of two COVID-19 waves in Fujian Province using a provincial multi-source surveillance system from week 49 of 2022 to week 29 of 2023. The main findings are as follows: ① The interval between the two observed epidemic peaks was 21 weeks (approximately 5 months). The epidemic transformed from a sharp-peaked first wave to a milder gradual-peaked second wave with substantially reduced peak levels, although differences in testing volume and surveillance conditions between waves warrant caution in interpreting cross-wave comparisons. ② Multi-source surveillance indicators were highly positively correlated, with the strongest correlation between sentinel hospital COVID-19 positivity rate and population nucleic acid positivity rate (rs = 0.92, *p* < 0.001); lagged cross-correlation analysis further indicated that these two indicators moved essentially in synchrony, without a consistent lead–lag relationship. ILI showed negative correlations with COVID-19 indicators exclusively in the descending phase of Wave 1. ③ Severe disease peaks lagged infection peaks by approximately 2 weeks. ④ The XBB variant required around 4 weeks from initial local detection to population predominance in this episode, which coincided with the peak of the second epidemic wave. ⑤ Slight differences existed in peak timing among different surveillance indicators, but overall trends were consistent.

The approximately 5-month interval between the two observed epidemic peaks is consistent with inter-wave intervals reported in previous multinational studies of Omicron-dominated COVID-19 epidemics (9–11). Immune escape of viral variants and natural waning of population immunity have been identified as core drivers of recurrent epidemic waves (12–16). Evidence has confirmed that the combined effect of immune escape mutations and antibody waning substantially reduces population immunity, promoting variant replacement and epidemic resurgence (13). Related studies also verified that these two factors constitute the dual core driving mechanism of multi-wave COVID-19 epidemics (15, 16). Nevertheless, because only two epidemic waves were observed in this study, the present data yield only a single inter-peak interval and cannot establish whether a recurrent epidemic cycle or periodicity exists; the observed interval should be regarded as a descriptive finding.

The markedly reduced peak levels and medical burden in the second wave should be interpreted with caution, because part of the observed reductions may reflect the dramatic decline in nucleic acid testing volume and changes in healthcare-seeking behaviour and reporting practices between the two waves, rather than epidemiological changes alone. With this caveat in mind, the reduction can be mainly attributed to two factors. First, herd immunity induced by BA.5.2 infection established a protective barrier (17–19). Studies have shown that natural infection with BA.5.2 elicits strong humoral and T-cell immune responses (IFN-γ) and cross-neutralization against variants such as XBB. Herd immunity generated by BA.5.2 infection in more than 80% of the Chinese population markedly reduced the severe case rate and medical burden during the XBB wave (18). A further study confirmed that BA.5 breakthrough infection provided protection against XBB.1.9.1 for at least 5 months, with 63% of individuals remaining free of reinfection and neutralizing antibodies maintained above protective thresholds, providing an immunological basis for the milder second wave (17). Second, the intrinsic pathogenicity of the XBB variant was attenuated (20, 21). Studies reported that the hospitalization rate for XBB/XBB.1.5 was only 17.0%, significantly lower than 21.3% for BA.5, with obvious reductions in severe illness, oxygen therapy requirement, and mortality, effectively alleviating medical pressure (20). A longitudinal study also confirmed that the XBB wave exhibited substantially lower infection and severe illness rates due to the dual effects of post-BA.5 immunity and antibody waning, which is highly consistent with the pattern of the second wave in this study (18). Additionally, the 2-week lag of severe case peaks behind community infection peaks identified in this study is consistent with international epidemiological evidence from European countries (22, 23). This spatiotemporal lag pattern provides a critical time window for proactive medical resource allocation and targeted severe case management, delivering practical guidance for epidemic preparedness.

Notably, the abnormal negative correlation between ILI proportion and COVID-19 surveillance indicators during the descending stage of the first wave reflects the staggered prevalence of multiple respiratory pathogens. The elevated ILI proportion represents the overall activity of acute respiratory infections rather than exclusive COVID-19 transmission, which introduces inherent confounding effects. Accordingly, the composite indicator combining ILI proportion and COVID-19 positivity rate cannot accurately reflect real-time COVID-19 prevalence, as non-SARS-CoV-2 respiratory pathogens may inflate ILI values and interfere with epidemic signal identification.

Although multi-source surveillance indicators showed slight differences in peak timing, their trends were highly correlated, reflecting distinct monitoring properties. Directly reported cases represented confirmed diagnoses and were strongly affected by holidays and reporting awareness. The fever clinic positive diagnosis ratio peaked 1 week earlier due to fluctuations in testing volume, but still reflected the underlying infection trend. Sentinel hospital positivity rate, supported by standardized sampling and stable coverage, showed the highest consistency with population nucleic acid positivity rate (rs = 0.92, *p* < 0.001) and may serve as a stable and efficient indicator for routine monitoring; however, the lagged cross-correlation analysis showed that it moved essentially in synchrony with population-level positivity rather than leading it, so its value as a proven early-warning tool remains to be validated in future studies with longer time series. For instance, during the first wave, the fever clinic positive diagnosis ratio peaked at week 51 of 2022, while fever clinic visits, population nucleic acid positivity rate, and sentinel hospital positivity rate all peaked at week 52. This discrepancy may be explained by a shift toward treatment-seeking behaviour at the peak epidemic phase, with reduced sampling and testing, leading to a spurious decrease in the positive diagnosis ratio rather than a true decline in infection level. This highlights the limitations of relying on a single indicator and supports the need for comprehensive interpretation using multi-source data.

The results of this study are consistent with overseas multi-wave surveillance studies, which also reported inter-wave intervals of roughly 4–5 months (9–11) and a lag of severe peaks behind infection peaks (22, 23). Studies from the United Kingdom (22) and Italy (23) support our observations regarding severe-case lag, and a US study reported a similar inter-wave interval pattern (24). Previous studies mostly emphasized the early warning value of single indicators. This study further examined the complementarity of multi-source surveillance indicators: during the mass nucleic acid screening phase, the population nucleic acid positivity rate reflected the proportion of positive results among tested individuals and complemented the other indicators; during routine prevention and control, the sentinel hospital positivity rate could serve as a relatively stable and efficient indicator in place of mass nucleic acid screening, while fever clinic data could capture rising trends at an early stage of epidemic upswings. The three types of indicators complement each other and effectively overcome the limitations of single indicators, providing practical evidence for optimizing surveillance systems; nevertheless, as discussed above, the early-warning capability of any single indicator should not be over-interpreted in the absence of validated predictive analyses.

*This study has several limitations*: ① The population nucleic acid testing volume declined dramatically between the two waves, from approximately 7.49 million tests at the first-wave peak to approximately 78,000 tests at the second-wave peak; shifts in testing policy, healthcare-seeking behaviour, reporting practices, and pre-existing population immunity across the two waves may explain part of the observed inter-wave differences, and comparisons of epidemic intensity or risk between waves should therefore be interpreted cautiously. ② The population nucleic acid positivity rate was calculated among tested individuals (community screening and voluntary/hospital-based testing) rather than a representative population sample; it reflects the test-positive proportion among those tested and cannot be directly equated to true population infection prevalence. ③ The fever clinic used a “positive diagnosis ratio” instead of a true positivity rate. This indicator was affected by the number of tests performed, and fluctuations in testing volume may have shifted peak timing, preventing precise reflection of actual infection levels. ④ The 1–2-week differences in peak timing among indicators resulted from discrepancies in monitoring definitions and data collection procedures (e.g., direct reporting for confirmed cases vs. fever clinic data for care-seeking patients), rather than inconsistent epidemic trends. ⑤ Some surveillance data were submitted as aggregated reports without automated capture via information systems, which may carry risks of manual interference or underreporting and affect data accuracy. ⑥ This study only analyzed two waves in Fujian Province; caution is needed when extrapolating results to other regions, especially given variations in population immunity and control measures that may influence wave intervals and epidemiological characteristics. ⑦ Numerous mild and asymptomatic infected persons do not visit medical institutions for testing, so these cases cannot be captured by hospital-based routine surveillance, resulting in possible underestimation of actual community infection scale and overall epidemic intensity. ⑧ Because only limited retrospective weekly data were available, the phase-stratified correlation analyses in some epidemic phases were based on small numbers of weekly observations, and formal predictive validation of the early-warning value of any single indicator was not feasible; these results should therefore be interpreted prudently.

*Future research can be optimized in the following aspects*: ① Promote the transition of fever clinic indicators from “positive diagnosis ratio” to standardized “positivity rate” by uniformly collecting “fever clinic visits – positive test counts” to eliminate the influence of testing volume and improve data accuracy. ② Strengthen information-based surveillance by implementing automated data capture using intelligent pre-processing software deployed in medical institutions nationwide (25), reducing manual intervention, enhancing timeliness and accuracy, and avoiding underreporting or misreporting associated with aggregated reporting. ③ Conduct multi-center and multi-region studies to verify inter-wave intervals and indicator correlations across regions with different immunity levels and control strategies. ④ Integrate viral variant surveillance data to further quantify the temporal association between variant emergence/replacement and epidemic peaks. Combined with research on immune waning and variant escape (13, 18), epidemic prediction models can be improved to enhance the foresight of epidemic prevention and control.

In conclusion, this study systematically characterizes the epidemiological evolution of two successive COVID-19 waves and comprehensively evaluates the practical performance of multi-source surveillance systems in real-world epidemic monitoring. The observed temporal epidemic patterns and differentiated indicator advantages provide empirical evidence for optimizing routine surveillance strategies and supporting timely, evidence-based prevention and control of future variant-driven respiratory infectious disease epidemics.

## 5 Conclusion

1. Two COVID-19 waves in Fujian were approximately 5 months apart, evolving from sharp peak to gradual peak with markedly reduced peak levels, although cross-wave comparisons should be interpreted with caution given the differing testing volumes and surveillance conditions.
2. Multi-source surveillance indicators were highly temporally consistent; lagged analysis showed that sentinel hospital and population-level indicators moved essentially in synchrony, supporting the complementary use of sentinel surveillance for routine monitoring.
3. Severe peaks lagged infection peaks by about 2 weeks, supporting timely medical preparedness.
4. The XBB variant reached predominance approximately 4 weeks after initial local detection in this episode; this descriptive observation cannot be generalized as a validated quantitative tool for predicting future variant-driven epidemic waves.

These findings support the optimization of surveillance systems and preparedness for multi-wave respiratory infectious diseases.

## Data Availability

The raw data supporting the conclusions of this article will be made available by the authors, without undue reservation.
